# Are submicroscopic chromosomal inversions predisposing factors for the t(9;22)(q34;q11.2) translocation in chronic myeloid leukemia?

**DOI:** 10.1186/s13039-015-0116-9

**Published:** 2015-02-22

**Authors:** Juan Ramón González García, Martín Daniel Domínguez Cruz, César Borjas Gutiérrez

**Affiliations:** División de Genética, CIBO-Instituto Mexicano del Seguro Social, Sierra Mojada # 800, Colonia Independencia, CP 44340 Guadalajara, Jalisco México; Doctorado en Genetica Humana, CUCS-Universidad de Guadalajara, Guadalajara, Jalisco México

**Keywords:** Chronic myeloid leukemia, t(9;22) translocation, Submicroscopic inversion, Duplicons, Non-allelic homologous recombination

## Abstract

A complex chromosomal rearrangement observed in a patient with chronic myeloid leukemia was explained as the consequence of a multistep process. The explanation involved an initial t(9;22) translocation with breakpoints distant from the *BCR* and *ABL1* genes followed by genomic deletions that produced the *BCR-ABL1* hybrid gene. We present an alternative model that fits the origin of the patient’s rearrangement better. The present model links submicroscopic inversions with the occurrence of the t(9;22) translocation and opens a new approach on the research on the disease.

## Letter to Editor

Saglio et al. advanced the involvement of duplicons in the initial interaction between chromosomes 9 and 22 leading to the t(9;22)(q34;q11.2) translocation in chronic myeloid leukemia (CML) [1]. They observed a complex chromosomal rearrangement in a patient with CML and explained it as consequence of a multistep process. Their explanation started with a t(9;22) translocation with breakpoints distant from the *BCR* and *ABL1* genes; then, specific genomic deletions occurring in a cell with such t(9;22) translocation produced the *BCR-ABL1* hybrid gene (Figure six *b* in [1]). Here, we present an alternative model that fits the origin of the patient’s rearrangement better and also opens a new focus on the research on the disease.

**Figure 1:**
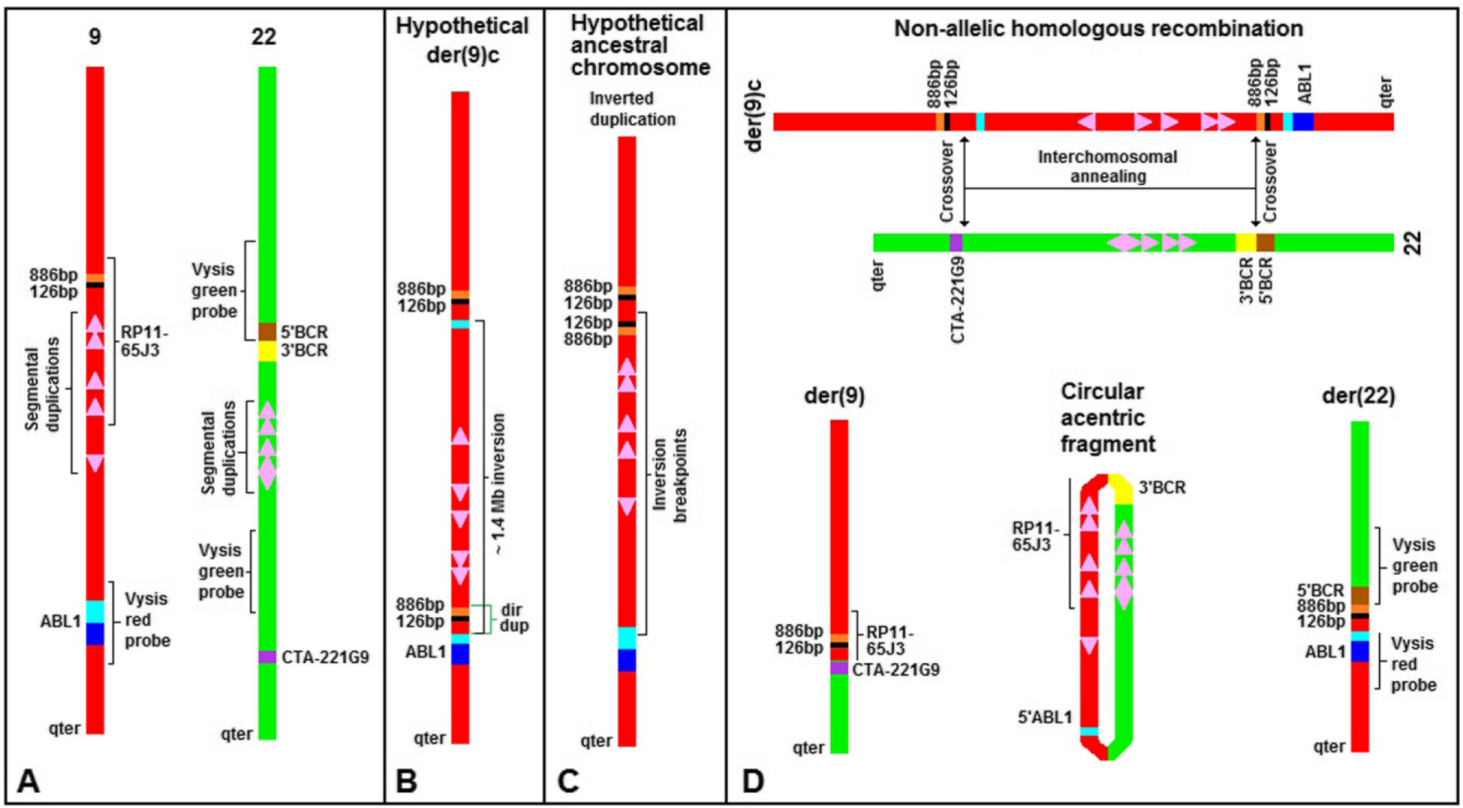
**Non-to-scale diagram to explain the chromosomal rearrangement observed by Saglio et al.** **[** 1 **].** **A)** Normal 9 and 22 chromosomes displaying some sequences of interest. Aqua box represents the *ABL1*-intron 1 sequence. Dark-blue box denotes the *ABL1* sequences downstream from intron 1. The depicted *BCR* gene sequences are split by the breakpoint. Duplicons were oriented according to information obtained from [2] and from the Database of Genomic Variants [http://dgv.tcag.ca/dgv/app/home (GRCh37/hg19)]. **B)** Proposed constitutional derivative chromosome 9 (der(9)c) carrying an inversion and a direct duplication. **C)** Hypothetical ancestral chromosome by which the proposed der(9)c could have been originated. **D)** Non-allelic homologous annealing − mainly at regions of duplicons − followed by recombination at specific points, renders the t(9;22) and a circular acentric fragment. For full explanation see text.

The clue supporting our proposal comes from their own data [1], which strongly support the presence of a partial duplication of the sequence covered by the BAC RP11-65J3, which maps at chr9:132090540–132244817 (^a^). The first of these duplicated segments is the one identified by sequencing the junction of the *BCR-ABL1* hybrid gene, which includes the 886 bp-126 base-pair (bp) segment [1]. As for the second duplicated segment, we infer that it is included in the sequence observed as a faint red signal on the der(9) (Figure two *b* in [1]). This inference is based on the finding of shared sequences on chromosomes 9 and 22, revealed by fluorescent in situ hybridization (FISH) with the BAC RP11-65J3 probe (Figure two *a* in [1]). That shared region, which is ~ 55 kilo-base (kb) long, spans the segments A’-B’, C’-D’, E’-F’, and G’-H’ on chromosome 9; and A-B, C-D, E-F, and G-H on chromosome 22, depicted in Figure two in [2] (^b^). If the faint red signal observed on normal chromosomes 22 (Figure two *a* in [1]) corresponds to a sequence of ~ 55 kb but could not be seen on the normal chromosome 22, in Figure two *b* [1], then it can be concluded that the faint red signal observed on the der(9), in the same Figure two *b*, must correspond to a sequence longer than 55 kb, which would therefore include the 886 bp-126 bp segment, located at ~ 10 kb proximal within the BAC RP11-65J3 sequence (Figure 1A) (^c^).

The specific reordering of sequences found by Saglio et al. in the der(22) [1], described by us according to the ISCN (2013) [3] as 22pter → *BCR*-intron14::886 bp-126 bp → ?::*ABL1-*intron1 → 9qter, calls for the union of the 886 bp-126 bp → ? duplicated segment with the *ABL1*-intron1 − in a direct orientation − before the occurrence of the t(9;22) translocation. This premise led us to hypothesize the existence, for that patient, of a constitutional derivative chromosome 9 (der(9)c), carrying two rearrangements: a direct duplication of part of the BAC RP11-65J3 sequence which includes the 886 bp-126 bp → ? segment, and an inversion of ~ 1.4 mega-base as shown in Figure 1B. Such der(9)c could result from a meiotic inversion in an ancestral chromosome − bearing an inverted duplication of part of the BAC RP11-65J3 sequence − with breakpoints being located in the *ABL1-*intron1 and within the duplicated segment of the BAC RP11-65J3 sequence (Figure 1C). Then, the t(9;22) translocation results from non-allelic homologous annealing of the duplicons located on each chromosome, subsequent recombination within such pairing structure, and a particular resolution of the Holliday junctions (Figure 1D). The der(22) generated in our model (Figure 1D) contains the *BCR-ABL1* fusion signal detected by FISH with the Vysis dual color probe and is also congruent with the specific reordering 22pter → *BCR*-intron14::886 bp-126 bp → ?::*ABL1-*intron1 → 9qter. On the other hand, the der(9) depicted in Figure 1D is consistent with both the faint red signal of BAC RP11-65J3 and the green signal of CTA-221G9 probe observed on the der(9) (Figure two *b* in [1]). The model also predicts that all deleted sequences shown in the Saglio’s et al. Figure three [1] presumably were lost as a circular acentric fragment (Figure 1D), a mechanism that can indeed account for most deletions related to the der(9). Moreover, the simultaneous occurrence of all these rearrangements, as predicted by our model, would better explain the absence of a more basic clone (i. e. the clone with the t(9;22) translocation but without deletion of *ABL1*-*BCR* sequences), both in the Saglio’s et al. patient and in others cases described elsewhere.

Finally, our contention is supported by some small constitutional inversions implicated as predisposing factors for chromosomal translocations [4,5]. Chromosomal inversions on 4p16 and 8p23 are related to the recurrent t(4;8)(p16;p23) translocation and were detected in 12.5% and 26% in non-affected control population [4]. Whether the der(9)c was really present in the Saglio’s et al. patient remains to be determined; however, there exists enough evidence in their work to hypothesize the involvement of an inversion which, on its own or in conjunction with the duplication, could be an etiologic factor of the t(9;22) translocation, in that case and, possibly, for most patients with CML.

## Endnotes

^a^Position of the BAC RP11-65J3 is not currently available in web-databases. We defined its position at chr9:132090540–132244817 taking in account positions of the sub-clones related with it: Ensembl Genes RP11-65J3.15 at chr9:132090540–132090680 - (ENST00000599815), ENST00000427109, ENST00000436710, ENST00000444125, ENST00000423122, ENST00000444641, ENST00000436510, ENST00000439014, ENST00000454408, and RP11-65J3.14 at chr9:132242884–132244817 - (ENST00000565882); [UCSC; http://genome.ucsc.edu/ (GRCh37/hg19)].

^b^Duplicons marked as A’-B’, C’-D’, E’-F’ and G’-H’ in the Albano’s et al. work [2] map at chr9:132231334–132236881 (~5.5 kb), at chr9:132208889–132218129 (~10 kb), at chr9:132155038–132186129 (~31 kb) and at chr9:132148664–132157375 (~8.7 kb), respectively [Database of Genomic Variants; http://dgv.tcag.ca/dgv/app/home (GRCh37/hg19)].

^c^The 126 bp exon maps at chr9:132101071–132101196 [UCSC; http://genome.ucsc.edu/ (GRCh37/hg19)].

## Abbreviations

bp: Base pair
CML: Chronic myeloid leukemia
FISH: Fluorescent in situ hybridization
kb: Kilo-base
